# Being Conscious of One’s Own Heroism: An Empirical Approach to Analyzing the Leadership Potential of Future CEOs

**DOI:** 10.3389/fpsyg.2018.02787

**Published:** 2019-01-17

**Authors:** Jose V. Pestana, Nuria Codina

**Affiliations:** ^1^Department of Social Psychology and Quantitative Psychology, University of Barcelona, Barcelona, Spain; ^2^Euncet Business School, Terrassa, Spain

**Keywords:** heroism, methodology, Myers-Briggs Type Indicator, leadership, interaction, psychological types

## Abstract

From the disciplinary field of the science of heroism, there is a need to deepen the processes that this science comprises, and at the same time, to test methods of inquiry to account for the variety of processes associated with this science. Linked to this sensitivity, the objective of this contribution is to jointly analyze, in a sample of future CEOs, what they imagine about heroism, their psychological types, and their values orientation. The sample consisted of 45 students (21 men and 24 women) between 22 and 47 years old (*M* = 26.69, *SD* = 4.47), who were part of a master’s program oriented toward training future CEOs to be leaders. The analytical instruments were the Myers-Briggs Type Indicator (MBTI), a story that each participant developed about him/herself as the main hero or heroine, and a questionnaire on personal values. In the psychological types observed, the functions of thinking and sensing predominated, with intuition and feeling residing at a lower level of consciousness. With regard to the stories, the majority of the sample offered tales in which the hero/heroine was confronted with a mystery to solve (or mission to fulfill), faced difficulties, and, finally, achieved harmony between the personal and the collective. Regarding the values, significant associations are observed between the gender, the characteristics of the psychological types, and the content of the story about their own hero / heroine. In sum, the research carried out offers an empirical approach to the study of the subjective elements of heroism, combining quantitative and qualitative aspects in an educational setting, and broadening the perspectives on the science of heroism.

## Introduction

The science of heroism has broadened the field of scientific explanations for leadership, due to the fact that it is not limited to unilaterally considering personal or social aspects as the main explanations for this process. Efthimiou and Allison (2017, p. 11) define heroism science as “a nascent multidisciplinary field which seeks to reconceptualize heroism as its correlates (the hero’s journey, heroic leadership, heroic imagination, everyday heroism, resilience, courage, altruism, etc.) through a close examination of the origins, types and processes of these interrelated phenomena.”

In fact, heroism science has reformulated the bases on which the main tendencies in the analysis of leadership have been established, that is: (1) the tendency that analyses leadership based on leaders’ personal characteristics; (2) the research on leadership that rests on the situation as an explanatory variable in this process; and (3) the most recent approaches that, starting from the notion of prototypicality and social identity, have gone beyond the one-directional analysis of the influence of the leader or the context, instead calling attention to what occurs between them.

After highlighting the contributions of heroism science in each of these three perspectives, we will examine heroism by considering the Analytical Psychology founded by C. G. Jung on the unconscious (personal, collective) and the psychological types – with values as a complementary indicator that shows the intersection between individual and social elements.

Introducing concepts of Analytical Psychology in the research of leadership as heroic accomplishment examines a perspective related to the foundational work by Campbell (1949/2004), offering an empirical approach to the study of the subjective elements of heroism (Allison et al., 2017) that combines qualitative and quantitative aspects. The qualitative data come from an *ad hoc* questionnaire where each participant told a story in which s/he was the main hero or heroine. The quantitative data come from two instruments: the Myers-Briggs Type Indicator (MBTI) and the Personal Values questionnaire. Albeit the MBTI is not universally accepted as a strong psychological measure, it is the only scale that the field of Analytical Psychology utilizes, and does so to explore the psychological types – the limitations and particularities of such testing is acknowledged and will be discussed later.

## Review of Literature

### Heroism Science. Broadening the Foundations of the Main Analysis on Leadership

#### The Leader, a Gifted Person?

Throughout its history, humanity has created leaders and caused them to emerge. This is demonstrated by the innumerable stories of gods, heroes, kings, great figures, and leaders who, depending on the case, have performed miracles, amazing feats, or successes (as well as meanness, stinginess, or failures). However, with the advance of the social sciences, a body of knowledge has been developed that, among other aims, tries to explain what makes leaders special. In this case, we are faced with a one-sided tendency that analyses leadership based on leaders’ personal characteristics – with what is known as charisma being the first.

The term charisma was used by Weber (1921/1946, p. 245) to highlight that: “The natural leaders in distress have been holders of specific gifts of the body and spirit; and these gifts have been believed to be supernatural, not accessible to everybody.” This specific characteristic of leaders was unrelated to historical and social circumstances – as it is a personal characteristic that few people have. After identifying leaders as gifted people with charisma – a term still commonly used outside the scientific field – studies were carried out on the personality of leaders (Judge et al., 2002; Bono and Judge, 2004; Neira Vaque et al., 2018; Zaccaro et al., 2018). These studies tried to define their profiles, aspiring to stimulate these traits in tomorrow’s leaders. Criticisms of this one-sided tendency focus on the little importance given to the effect of a certain historical moment on the profile of leaders. For example, consider historical figures in the past who, although leaders in their time, today would be rated negatively due to their ideas, behaviors, or both.

The incorporation of heroism into the explanation of leaders’ behavior has shown that the individual basis for what is heroic encompasses not only a certain personality profile, but also a series of features (categories, prototypes, self-representations: Shahar, 2013; Kinsella et al., 2015b; Israeli et al., 2018) that can be learned or fostered (or both). Moreover, heroes have been known to fulfill a series of functions that can be a powerful social influence for other individuals (Kinsella et al., 2015a). There is no doubt about the knowledge enrichment stemming from conceiving the leader as a hero; however, this analysis has also led to revisiting the importance of the context in shaping heroic leaders.

#### From the Person to the Environment: The Context of the Leader

Focusing on what leaders do and the way their actions are received by their followers, psychosocial knowledge has an undisputable point of departure in two classic studies: the one by Lewin and Lippitt (1938) and the one by Lewin et al. (1939). In these studies, six groups of 10- and 11-year-old boys experienced three leadership styles – autocratic, democratic, and *laissez-faire* – in order to demonstrate the effect of each style on the atmosphere and productivity of the groups, in addition to showing the subordinates’ flexibility and ability to respond to their leaders’ proposals. These experiments were fundamental in the development and consolidation of the other one-sided perspective in the research on leadership: the one that rests on the situation as an explanatory variable in this process.

After these pioneer researches, studies were developed that were oriented toward defining which contextual variables would favor leadership (Fiedler, 1964; Hill, 1969; Ayman et al., 1995). However, as far as leadership is concerned, an explanation that focuses exclusively on the context was not free of criticism (Graeff, 1997). In fact, this one-sided tendency involved a risk that was synthesized in the image of the film character Forrest Gump: the risk that anyone, regardless of his/her personal deficiencies, could become a leader if called upon to perform specific tasks at a certain moment in time.

From heroism science, the context has acquired a double value with regard to leadership: as a social situation that favors heroic behaviors and as an educational setting that foments heroism in day-to-day life. In fact, “the heroic learner’s journey is an inspirational tool that shows how a learner could undergo transformation in the field of education. The educational transformation of the heroic learner can benefit all members of society, including at-risk learners” (Pascoe, 2018, p. 63). Thus, the context in which the leader is shaped and develops must try to foment an approximation to the idea of “heroism as embedded and embodied in the everyday” (Efthimiou, 2017, p. 158), as the transformation process of the hero – which is particular in each case: Allison and Goethals (2017).

#### Between the Personal and the Social Realms: Leadership as Transformational and Identitary

In more recent years, interpersonal relationships in the leadership process have been acquiring greater relevance. Specifically, it is no longer a question of performing a one-directional analysis of the influence of the leader or the context, but rather of calling attention to what occurs between them. Examples of this can be found in the theories on transformational/charismatic leadership and social identity theory.

Transformational leadership has reformulated the idea of charisma – or the attraction exerted by a person: Lowe et al. (1996), Avolio and Bass (1999), and Sun et al. (2017). Unlike the conception of charisma as a gift or virtue of leaders – as stated by Weber (idem) – this proposal understands charisma to be an attribution made by the followers. Thus, the followers will consider their leader to be charismatic to the extent that s/he: emphasizes the importance of the effort made and its symbolic value; increases the expectations about achieving the objectives and the importance of reaching the proposed goal; transmits faith in a better future; and makes a personal commitment, leading by example (Molero, 2011). Moreover, transformational leadership is a style that predicts behavioral outcomes – such as task performance – and attitudinal outcomes in the subordinates (such as creativity, organizational commitment, or empowerment, among others: Banks et al., 2016).

The centrality of interpersonal relationships, consubstantial to transformational / charismatic leadership, has increased its explanatory power with the incorporation of the basic proposals of the two most influential theories in European social psychology: social identity theory (proposed by H. Tajfel) and self-categorization theory (whose main representative has been J. C. Turner) – Hogg (2001) and Haslam et al. (2011a,b). According to these theories, leadership is understood as a process in which it is fundamental to belong to a group and feel that this belonging is important to our self-perception.

Thus, effective leadership is conceived as the result of: considering the leader to be part of the group s/he represents; the leader’s defense of the group, as far as s/he is just another member (although perhaps the most prototypical); reminding the group who they are and where they are headed; and creating a future scenario that responds to the group identity (Haslam et al., 2017). These characteristics are summarized in the conception that “effective leadership is always an *identitary leadership*” (Haslam et al., 2011a: 108 —original emphasis).

With the reinterpretation of leadership in terms of social identity, there is a balance between personal and situational components in the analysis of the phenomenon, which should not be confused with its stability. In this regard, Haslam et al. (2011a) insists on the processual and transitory nature of leadership, which simply means accepting that the person who leads the group today may be substituted by another member of the group tomorrow. In our opinion, this nuance about its transitory nature adds a characteristic to identitary leadership that is not explicitly stated in previous contributions: the fact that one theory contemplates why a person comes to occupy a position of leadership, as well as the way the leader stops being one.

The incorporation of identity in the analysis of leadership has represented an advance that includes both this process and Social Identity Theory, on which it is based. However, the dynamic leader-follower identity interplay remains relatively unexplored (Epitropaki et al., 2017; Kramer, 2017). With regard to the idea of prototypicality, the studies by Shahar (2013) and Kinsella et al. (2015a,b), mentioned above, warned about the complexity of the traits and functions of the hero.

Based on the idea of the depth – individual and social – that heroism can reach in everyday life, our study incorporates Analytical Psychology. This perspective, related to the foundational study by Campbell (1949/2004), offers theoretical and empirical elements that can contribute developments on the well-known subjective aspect of heroism (Allison et al., 2017).

### Leadership as a Heroic Accomplishment: A Contribution From Analytical Psychology

In the Analytical Psychology founded by Jung (1952/1976, §251), the hero is a basic figure of the collective unconscious, that is, of the repository of symbolic contents that accompany humanity throughout its history: “The finest of all symbols of the libido is the human figure, conceived as a demon or hero (§251)… What we seek in visible human form is not man, but superman, the hero or god, that *quasi-human* being who symbolizes the ideas, forms and forces which grip and mold the soul (§259 – original emphasis).” This conception places Analytical Psychology among the orientations that understand that “mental images and conceptions of heroism are... hardwired into us” (Allison et al., 2017, p. 9).

For Jung (1959/1980, §284), in the different stories that describe the hero’s adventures, whose core and characteristics were synthesized by Campbell (1949/2004 – see Table 1), the “main feat is to overcome the monster of darkness: it is the long-hoped-for and expected triumph of consciousness over the unconscious.” In this regard, every story whose plot has mobilized psychic energy from early times reveals those contents that, from the collective unconscious, are connected to the personal unconscious.

**Table 1:** Contents of the hero/heroine story (based on Campbell, 1949/2004).

Contents	
**Departure**	
The Call to Adventure	Appearance of a mystery that must be solved.
Refusal of the Call	Difficulties in giving up one’s own interests when faced with the mission to be fulfilled.
Supernatural Aid	Appearance of a protector figure (and his/her lucky charms, amulets) to help at the beginning of the mission.
The Crossing of the First Threshold	First step taken within the sacred area.
The Belly of the Whale	Staying in the sphere of rebirth ormetamorphosis.
**Initiation**	
The Road of Trials	Facing difficulties.
The Meeting with the Goddess	Appearance of a presence that announces everything that can be known about the mission.
Woman as the Temptress	Appearance of a presence that can interfere with the mission to becarried out.
Atonement with the Father	Having a transcendent vision after accepting what is one’s own.
Apotheosis	Divine state after overcoming ignorance.
The Ultimate Boon	Adventure that the chosen one carries out with ease.
**Return**	
Refusal of the Return	Rejection of returning with the trophy won.
The Magic Flight	Flight with miraculous obstacles.
Rescue from Without	Call from the Society to re-join it.
The Crossing of the Return Threshold	First step taken in starting the return trip.
Master of the Two Worlds	Transition between the world or dimensions of the universe known before and during the mission.
Freedom to Live	Reconciliation between the individual conscience and the universal will, that is, integrating the achievements of the mission in the original context.


The need to integrate the contents of the unconscious (collective, personal) into the conscious is precisely what underlies the active imagination, “a sequence of fantasies produced by deliberate concentration” (Jung, 1959/1980, §101). From the contemplation of some fragment that is significant to the person, a sudden awareness can occur about a psychic content that had previously been ignored. This awareness can be observed through the analysis of stories: both in stories that have transcended to different periods (von Franz, 1980, 1995, 1997), as well as in current narratives about heroism that mobilize psychic processes in those who read them (Sanders and van Krieken, 2018). This psychic movement can be explained by the two functions that the hero stories fulfill (Allison and Goethals, 2014, p. 170): the *epistemic* function (“the knowledge and wisdom that hero stories impart to us”) and the *energizing* function (“the ways that hero stories inspire us and promote personal growth”).

However, it should be noted that each individual becomes conscious depending on his / her psychic activity, known as psychological types. When describing the types, the main distinction Jung (1921/1976) makes is the one corresponding to extraversion and introversion. Both make up various adaptive relationships based on the way the subject and object relate to each other. In the conscious attitude related to *extraversion*, it has been observed that the “orientation by the object predominates in such a way that decisions and actions are determined not by subjective views but by objective conditions” (Jung, 1921/1976, §563). By contrast, the extraverted unconscious attitude includes subjective impulses that are not recognized. In the case of introversion, it “is naturally aware of external conditions, [but] selects the subjective determinants as the decisive ones… Whereas the extravert continually appeals to what comes to him from the object, the introvert relies principally on what the sense impression constellates in the subject” (Jung, 1921/1976, §621). In this case, the introverted unconscious attitude is characterized by an intensification of the object.

In addition to the extraversion-introversion distinction, there are four psychological functions, two rational (thinking, feeling) and two irrational (sensing, intuition):

– *Thinking* (T), “following its own laws, brings the contents of ideation into conceptual connection with one another” (Jung, 1921/1976, §830).
– *Feeling* (F) includes two processes: on the one hand, attributing to an object “a definite value in the sense of acceptance or rejection (‘like’ or ‘dislike’)”; on the other hand, experiencing a mood that also implies “a valuation; not of one definite, individual conscious content, but of the whole conscious situation at the moment, and, once again, with special reference to the question of acceptance or rejection” (Jung, 1921/1976, §724).
– Without being held to the laws of reason, *sensing* (perception, S) “mediates the perception of a physical stimulus… [and] is related not only to external stimuli but to inner ones, i.e., to changes in the internal organic processes” (Jung, 1921/1976, §792).
– And, finally, *intuition* (I)
  “mediates perceptions in an *unconscious way*. Everything, whether outer or inner objects or their relationships, can be the focus of this perception... In intuition a content presents itself whole and complete, without our being able to explain or discover how this content came into existence” (Jung, 1921/1976, §770 – original italics).

An example that synthesizes the predominance of one psychological function or another can be found in Guggenbühl-Craig (1980/2008, p. 68 – italics added):

“A *sensation* type is affected by the color of a woman’s skirt. A *feeling* type is depressed and irritable in the same woman’s presence. An *intuitive* [type] suspects that the woman has just criticized him to a mutual friend, while a *thinking* type considers why it is that the woman blushed slightly on seeing him.”

The instrumentalization of this typology has been made possible through the application of the MBTI (Briggs Myers, 1985/2000; a historical review of the development of this instrument at: https://www.myersbriggs.org/). This scale offers data on polarities related to introversion-extraversion, thinking-feeling, and sensation- intuition; moreover, the MBTI incorporates judging (J) and perceiving (P) as attitudes to approach the outside world. From the combinations of these aspects, a total of 16 profiles are derived that can vary throughout life, with their comprehension leading to better self-understanding (Briggs Myers and McCaulley, 1985; Briggs Myers, 1993).

The accumulated scientific evidence based on this instrument includes arguments in favor of and against its use. Supporting the MBTI, Lloyd (2015) highlights the importance of its structure for pairs or polarities – consistent with its Jungian base – compared to other nomenclatures that can lead to biased ratings due to social desirability (regarding certain personality factors that are considered positive); in the words of Furnham (1996), the issue would be to warn and take into account the differences among the conceptions of personality in terms of types and traits, respectively. With regard to criticisms of the MBTI, they usually come from those who defend the use of the instrument supporting the Five-Factor Model of Personality (FFM: McCrae and Costa, 1989), which is an equivalent open-source assessment-tool that scholars are free to use and cite, with numerous contributions certifying its suitability in the study of the personality (Cooper et al., 2017).

Between these two extremes, some authors suggest accompanying the administration of the MBTI with other scales, in order to prevent the possible weaknesses of this instrument (Persky et al., 2015). In our case, in the present study, we undertake the analysis of the psychological types along with the analysis of values – as a concept that shows the degree to which an individual is linked to what is socially predominant in his/her time. This particular aspect of values is similar to the different psychological types, given that for each individual it is possible to contemplate different forms of identification and awareness of the elements of the collective unconscious.

Values are cognitive representations that respond to needs transformed into goals (Schwartz et al., 2001), guiding human actions (Gouveia et al., 2015). In addition to this, values are sensitive to historical and cultural aspects. For example, at certain times, there may have been a predominance of individualistic (with values such as Achievement, Power, Self-direction, Stimulation and Hedonism), collective (Conformity, Tradition, Benevolence), or mixed (Universalism, Security) interests. With regard to leadership, the importance of values has been shown (Schwartz and Sagie, 2000; Krishnan, 2001; Sosik, 2005; Nader and Castro, 2007, 2009), although they have only been partially analyzed and in specific settings such as military institutions.

From what is presented here – and to synthesize – we can highlight the two main ideas underlying this study:

• The science of heroism, in spite of being in its early stages, has contributed alternative and novel interpretations to the main tendencies in the analysis of leadership, such as the personal attributes of the leader, the social situations that favor certain leaderships, or the ways leaders and followers relate to each other (linked mainly to identitary phenomena).
• Due to its interstitial nature between the individual and social realms, leadership as heroic accomplishment is susceptible to being analyzed from Analytical Psychology, in particular, based on the relationships between the psychological types, the collective imagination about heroism, and the value orientations. This incorporation makes it possible to approach deeper content that can support what is heroic in the daily lives of leaders.

Based on these ideas, this research has the objective of jointly analyzing, in a sample of future CEOs, what they imagine about heroism, their psychological types, and their values orientation. In fact, considering a sample of future CEOs is relevant given that this role can integrate leadership and heroism (Decter-Frain et al., 2017), with a sphere of influence that can range from the work team, which is coordinated with the rest of an organization – and, as the case may be, reaching an impact on society as a whole. By doing so, we aim to contribute a vision of leadership that, similar to the idea of wholeness as an attribute of heroism (Efthimiou et al., 2018), broadens the base of this emerging science.

## Materials and Methods

### Participants

Participants were 45 students (21 men and 24 women) between 22 and 47 years old (*M* = 26.69 years; *SD* = 4.47), who were part of a master’s program oriented toward training future CEOs as leaders. Participation in the study was voluntary, i.e., no payment or academic rewards (such as extra marks for subjects or course credits) were given.

### Instruments

Data were collected using three instruments, along with the necessary demographics: the MBTI, an *ad hoc* questionnaire where each participant told a story in which s/he was the main hero or heroine, and the Personal Values questionnaire (adaptation of the Schwartz Value Survey to the Spanish-speaking context). This combination of instruments integrates qualitative and quantitative data, and so it offers a holistic view of the subjective aspects of leadership as heroic accomplishment.

#### Myers-Briggs Type Indicator

This questionnaire corresponds to the Spanish version of form G with 126 items (117 items with two response options, and the other nine with three) from the test that specifies the psychological typology developed by C. G. Jung. It considers the distinction between extraversion-introversion and the four functions of the psyche: thinking (T), feeling (F), sensation (S), and intuition (N). Depending on the person’s degree of awareness of each of these functions, they are considered primary, auxiliary, tertiary, and lower functions. In addition to what was presented above about the MBTI as an instrument to analyze the psychological types, the suitability and validity of the MBTI – particularly for the case at hand – have been documented in studies that have used it to analyze leader profiles (Brown and Reilly, 2009; Mattare, 2015), student typologies (Carlson, 1985; Ribeiro Filho et al., 2010; Bak, 2012; Ihm et al., 2017; Mohammadi et al., 2018), and even learning styles (Daisley, 2011). In the present study, the information obtained was analyzed according to the prevalence of the 16 psychological types, as well as the four axes (or main characteristics) on which these types are organized: focusing the attention (extraversion-introversion), making decisions (thinking-feeling), orientation toward the outer world (judging-perceiving), and taking in information (sensing-intuition).

#### Story About One’s Own Hero/Heroine

This *ad hoc* questionnaire consisted of writing a story in which the respondent was the protagonist. Responses were told to include a beginning, a development (rising and falling action), and an end (denouement), but they were free to choose the topics and the degree of realism of the story elements, in order to obtain an approximation to the degree to which heroism could be articulated – with a greater or lesser degree of consciousness – into the life narratives (Walker, 2017). In other words, the hero /heroine could, for example, have special powers or have his/her adventures in different eras or contexts. The stories were analyzed according to the characteristics described by Campbell (1949/2004) regarding the three core parts of the hero’s adventure: *Departure* (The call to adventure, Refusal of the call, Supernatural aid, The crossing of the first threshold, The belly of the whale); *Initiation* (The road of Trials, The meeting with the goddess, Woman as the temptress, Atonement with the father, Apotheosis, The ultimate Boon); and *Return* (Refusal of the return, The magic flight, Rescue from without, The crossing of the return, Threshold, Master of the two worlds, Freedom to live). Specifically, for each core part of the participant’s story, its predominant characteristics were identified. For example: if the part corresponding to the Departure gives more weight to the Refusal of the call, this characteristic was awarded a frequency of 1 (and so on for the other two core parts of the story – Initiation and Return). Table 1 summarizes the contents of the core parts and their characteristics.

#### Personal Values Questionnaire

This instrument is the Spanish adaptation of the Schwartz Value Survey (Schwartz, 1992), carried out by Abella García et al. (2017) based on the suggestions of Schwartz and Boehnke (2004). This version consists of 50 items that evaluate the following 10 values (five items per value): Tradition, Security, Stimulation, Conformity, Hedonism, Achievement, Benevolence, Power, Self-direction, and Universalism. Each item can be scored from 1 (“I have it very little or not at all”) to 7 (“I have it a lot or to a high degree”). The score for each value is obtained by adding up the points on the five items, yielding a range between 5 (in the case of giving the lowest score to the five value items) and 35 points (if all the items in the same value were given a score of seven). The results obtained were analyzed using descriptive statistics and Student’s *t-*test.

### Procedure

Data collection was carried out in paper format, before class time – in order to avoid interfering with the participants’ master’s degree classes – and in person. It took three sessions (one per instrument). The information was collected during the Fall Semester of the 2017–2018 academic year, beginning 1 month after classes started and ending 15 days before the final exams. This timing was important: it was intended to prevent contagion by outside variables such as extra academic work, tiredness, and class absenteeism. Data collection was carried out by members of the research team who were previously trained in the application of the set of research instruments. With the consent of the teaching staff at the center, and after the research project had been introduced, the students filled out the instruments voluntarily after signing the informed consent. In each data collection session, the time required to fill out the instrument was less than 1 h. Confidentiality of the data was guaranteed, and the data were processed using version 24 of the SPSS program.

## Results

The data obtained in this study refer to the predominant psychological types, the main characteristics of the stories of the participants as heroes / heroines, and the orientation in terms of personal values.

### Psychological Types

The analysis of the psychological types (Table 2) shows the predominance of two types over the others: the ESTJ profile (Extraverted Thinking with Introverted Sensing: *n* = 9, equivalent to 25.7% of the participants) and the ISTJ profile (Introverted Sensing with Extraverted Thinking: *n* = 7, representing 20.0% of the sample). Both profiles share sensing and thinking as dominant and auxiliary functions: in the case of the ESTJ profile, thinking is the dominant function, and sensing is auxiliary – contrary to what occurs in the ISTJ profile. This same relationship is observed in the tertiary and lower functions, which are Intuition and Feeling, respectively, for ESTJ (in the inverse order for ISTJ). As the main differential trait between these profiles, it can be observed that whereas the focus of attention of the ESTJ is extraverted, in the case of the ISTJ it is introverted.

**Table 2:** Percentage of participants according to their Psychological Types.

Psychological types	*n*	%
ISTJ / Introverted Sensing with Extraverted Thinking	7	20.0
ISFJ / Introverted Sensing with Extraverted Feeling	0	–
ESTP / Extraverted Sensing with Introverted Thinking	3	8.6
ESFP/ Extraverted Sensing with Introverted Feeling	2	5.7
INTJ / Introverted Intuition with Extraverted Thinking	2	5.7
INFJ / Introverted Intuition with Extraverted Feeling	0	–
ENTP / Extraverted Intuition with Introverted Thinking	2	5.7
ENFP / Extraverted Intuition with Introverted Feeling	0	–
ISTP / Introverted Thinking with Extraverted Sensing	3	8.6
INTP / Introverted Thinking with Extraverted Intuition	3	8.6
ESTJ / Extraverted Thinking with Introverted Sensing	9	25.7
ENTJ / Extraverted Thinking with Introverted Intuition	2	5.7
ISFP / Introverted Feeling with Extraverted Sensing	0	–
INFP / Introverted Feeling with Extraverted Intuition	0	–
ESFJ / Extraverted Feeling with Introverted Sensing	1	2.9
ENFJ / Extraverted Feeling with Introverted Intuition	1	2.9


Considering the psychological types according to their four main basic characteristics (Table 3), the highest frequencies are observed: in focusing the attention, Extraversion (*n* = 20, 57.1%); in making decisions, Thinking (*n* = 31, 88.6%); in orientation toward the outer world, Judging (*n* = 22, 62.9%); and in taking in information, Sensing (*n* = 25, 71.4%). The differentiation of these tendencies based on gender shows significant associations (information not tabulated) for taking in information (χ^2^ = 5.53, *p* = 0.019). Specifically, the percentage of men is 52.9% for the sensing type and 47.1% for the intuitive type; however, for the women, these percentages are 88.9 and 11.1%, respectively.

**Table 3:** Percentage of participants according to the basic characteristics of the Types.

Characteristics of the Types	*n*	%
**Focusing the attention**	
Extraversion	20	57.1
Introversion	15	42.9
**Making decisions**		
Thinking	31	88.6
Feeling	4	11.4
**Orienting toward the outer world**		
Judging	22	62.9
Perceiving	13	37.1
**Taking in information**		
Sensing	25	71.4
Intuition	10	28.6


### Stories About Their Own Hero/Heroine

With regard to the stories described by the participants, in which they were the leading heroes / heroines (Table 4), a tendency predominates in each part of the story. Specifically, Departure is mainly characterized by The Call of Adventure (*n* = 23, 65.7%), Initiation by The Road of Trials (*n* = 24, 68.6%), and Return by Freedom to Live (*n* = 19, 54.3%).

**Table 4:** Percentage of participants according to the main tendencies in each part of the hero/heroine story.

Part of the hero/heroine story	*n*	%
**Departure**	
The Call to Adventure	23	65.7
Refusal of the Call	5	14.3
Supernatural Aid	4	11.4
The Crossing of the First Threshold	0	–
The Belly of the Whale	2	5.7
N/A	1	2.9
**Initiation**		
The Road of Trials	24	68.6
The Meeting with the Goddess	2	5.7
Woman as the Temptress	1	2.9
Atonement with the Father	1	2.9
Apotheosis	4	11.4
The Ultimate Boon	0	–
N/A	3	8.6
**Return**		
Refusal of the Return	1	2.9
The Magic Flight	0	—
Rescue from Without	0	—
The Crossing of the Return Threshold	4	11.4
Master of the Two Worlds	7	20.0
Freedom to Live	19	54.3
N/A	4	11.4


As examples of each of the predominant tendencies in each part of the stories collected, we present the following (the extension of the text tries to situate the reader in the context of the story, highlighting in italics the fragment that illustrates the corresponding tendency):

#### The Call of Adventure/Departure

“It is a winter night. A group of fishermen and their leader were on the open sea doing their work. It was the third night of their journey, and everything was going very well, just as planned.

The problem arose on the fifth night, when an accident on the boat caused technical damage … *the question was whether to risk the lives of the fishermen and fulfill their mission satisfactorily – as they had been doing, or to return, having failed in their task. It was in this situation where the leader developed his/her skills.*”

#### Initiation/the Road of Trials

“R was a young man who had the gift of changing the size of things just by thinking about it …One day they arrived at a place where a witch, seeing happy people, looked for the reason, and she cast a spell whereby, instead of changing the size, things would disappear by making them very small. *After thinking a lot about how to use the spell to his advantage, R thought that perhaps by reducing the size of the witch, her powers would no longer work, until disappearing completely. But because he did not know if this would work, he did not want to risk annoying the witch.*”

#### Return/Freedom to Live

“Immediately, the aliens landed on the Earth, kidnapping the bad guys and explaining to humanity the truth and hidden secret throughout history. *Humanity, thanks to this, entered a cycle of illumination and creation of better futures.*”

For the tendency found in each part of the story, we went on to dichotomize the categories in the following way: in Departure, The Call of Adventure-Other departures; in Initiation, The Road of Trials-Other initiations; and in Return, Freedom to Live-Other returns. This recategorization made it possible to observe significant associations between the contents of the stories about their own hero / heroine and the predominant values of the participants.

### Values

Regarding values (Table 5), the highest scores are observed on Benevolence (*M* = 29.51, *SD* = 3.59), Self-direction (*M* = 28.94, *SD* = 3.36), and Conformity (*M* = 28.20, *SD* = 3.51). By contrast, the values with the lowest scores were Power (*M* = 24.51, *SD* = 4.36), Universalism (*M* = 25.66, *SD* = 4.33), and Tradition (*M* = 25.11, *SD* = 3.65). Comparing these tendencies by gender (Table 5), significant differences were obtained on Tradition (*t* = -2.36, *p* = 0.024), Conformity (*t* = -2.28, *p* = 0.029), and Universalism (*t* = -2.34, *p* = 0.025); in all cases, the women had higher scores than the men.

**Table 5:** Types of values. Differences according to gender.

Values	Total sample (*n* = 35)		Men (*n* = 17)		Women (*n* = 18)		*t*	*p*
	*M*	*SD*	*M*	*SD*	*M*	*SD*		
Tradition	25.11	3.65	23.71	3.31	26.44	3.53	–2.36	0.024
Security	26.91	3.35	26.24	3.53	27,56	3,485	–1.17	0.250
Stimulation	26.14	4.25	26,06	3,864	26,22	4,710	–0.11	0.911
Conformity	28.20	3.51	26.88	4.09	29.44	2.35	–2.28	0.029
Hedonism	28.06	3.76	27.76	4.35	28.33	3.21	–0.44	0.662
Achievement	27.91	4.19	28.12	5.03	27.72	3.35	0.27	0.785
Benevolence	29.51	3.59	28.76	3.15	30.22	3.91	–1.20	0.236
Power	24.51	4.36	24.53	5.00	24.50	3.82	0.02	0.984
Self-direction	28.94	3.36	28.65	3.75	29.22	3.04	–0.49	0.623
Universalism	25.66	4.33	24.00	4.10	27.22	4.03	–2.34	0.025


*Values, means on a scale of 7 to 35 (see section “Materials and Methods”).*

In addition to the gender differences, the emphasis on certain values was also observed depending on the main characteristics of the psychological types (Table 6). Specifically, Achievement obtained higher scores in those with a predominance of Thinking (*t* = -2.71, *p* = 0.011), Hedonism was greater in those with a predominance of Perceiving (*t* = -2.76, *p* = 0.009), and Benevolence was stronger in profiles corresponding to Judging (*t* = 2.23, *p* = 0.033) and Sensing (*t* = 2.24, *p* = 0.036). Finally, the values of Power (*t* = 1.85, *p* = 0.073) and Self-Direction (*t* = 2.07, *p* = 0.047) obtained higher scores in participants who, in their story about their own hero / heroine, wrote a Return with a predominance of Freedom to Live content (information not tabulated).

**Table 6:** Types of values. Differences according to the main characteristics of the Psychological Types.

			Focusing the attention						Making decisions					
	Total sample (*n* = 35)		Extraversion (*n* = 20)		Introversion (*n* = 15)		*t*	*p*	Thinking (*n* = 31)		Feeling (*n* = 4)		*t*	*p*
Values	*M*	*SD*	*M*	*SD*	*M*	*SD*			*M*	*SD*	*M*	*SD*		
Tradition	25.11	3.65	25.40	3.50	24.73	3.93	–0.52	0.601	25.00	3.70	26.00	3.55	–0.51	0.614
Security	26.91	3.35	26.85	3.04	27.00	3.83	0.12	0.898	26.94	3.54	26.75	1.50	0.18	0.855
Stimulation	26.14	4.25	26.35	4.64	25.87	3.81	–0.33	0.738	25.97	4.24	27.50	4.72	–0.67	0.506
Conformity	28.20	3.51	28.05	3.73	28.40	3.31	0.28	0.775	28.10	3.68	29.00	1.82	–0.80	0.450
Hedonism	28.06	3.76	27.65	4.08	28.60	3.35	0.75	0.456	28.26	3.60	26.50	5.19	0.87	0.387
Achievement	27.91	4.19	28.40	3.84	27.27	4.68	–0.78	0.437	28.55	3.84	23.00	3.91	2.71	0.011
Benevolence	29.51	3.59	29.80	3.69	29.13	3.54	–0.54	0.592	29.68	3.47	28.25	4.78	0.74	0.463
Power	24.51	4.36	25.48	3.98	23.27	4.68	–1.48	0.146	24.90	4.11	21.50	5.80	1.49	0.145
Self-direction	28.94	3.36	28.40	3.76	29.67	2.71	1.10	0.277	29.29	3.28	26.25	3.09	1.74	0.090
Universalism	25.66	4.33	25.20	4.06	26.27	4.74	0.71	0.479	25.84	4.45	24.25	3.30	0.86	0.430

			**Orienting toward the outer world**						**Taking in information**					
	**Total sample** **(*n* = 35)**		**Judging** **(*n* = 22)**		**Perceiving** **(*n* = 13)**		***t***	***p***	**Sensing** **(*n* = 25)**		**Intuition** **(*n* = 10)**		***t***	***p***
**Values**	****M****	***SD***	***M***	***SD***	***M***	***SD***			***M***	***SD***	***M***	***SD***		

Tradition	25.11	3.65	25.73	3.75	24.08	3.35	1.34	0.190	25.08	3.74	25.20	3.61	–0,08	0.931
Security	26.91	3.35	27.32	2.85	26.23	4.10	0.92	0.362	27.24	3.12	26.10	3.92	0.90	.372
Stimulation	26.14	4.25	25.27	4.35	27.62	3.79	–1.66	0.106	25.40	4.29	28.00	3.74	–1.77	0.091
Conformity	28.20	3.51	28.77	2.81	27.23	4.41	1.26	0.214	28.76	3.00	26.80	4.41	1.52	0.138
Hedonism	28.06	3.76	26.95	4.06	29.92	2.29	–2.76	0.009	27.84	3.18	28.60	5.10	–0.53	0.597
Achievement	27.91	4.19	28.41	3.69	27.08	4.97	0.90	0.372	28.36	3.56	26.80	5.53	0.99	0.328
Benevolence	29.51	3.59	30.50	3.24	27.85	3.64	2.23	0.033	30.28	3.56	27.60	3.02	2.24	0.036
Power	24.51	4.36	24.86	4.54	23.92	4.15	0.62	0.537	24.76	4.40	23.90	4.43	0.51	0.610
Self-direction	28.94	3.36	28.82	3.40	29.15	3.43	–0.28	0.781	23.90	4.43	29.80	3.45	–0.95	0.349
Universalism	25.66	4.33	26.41	4.14	24.38	4.50	1.35	0.185	26.44	3.80	23.70	5.12	1.74	0.091


*Values, means on a scale of 7 to 35 (see section “Materials and Methods”).*

## Discussion and Conclusion

The results obtained offer an empirical base that considers three aspects together: the psychological typology of potential leaders, the basic characteristics of their images of heroism, and the personal values that orient them. Specifically, this process has been investigated in an educational setting where heroic leaders of the future can be shaped.

The psychological types observed – in particular, the predominance of the functions of thinking and sensing, with intuition and feeling being at a lower level of consciousness – can orient the training of future leaders. Following the characterization by Jung (1921/1976), in the case at hand, we have professionals whose psyche devotes its energy to conceptually structuring what is perceived – whether through extraversion or introversion. Consequently, this situation entails the need to make conscious the feelings that accompany life experiences, while attending to the contents that represent alternative images of reality – that is, intuition. From the perspective of Analytical Psychology, our findings can complement analyses such as the one carried out by Samuels (2000, p. 40) with regard to the good-enough leader: a person who, aware of his/her limitations, forms a constellation of archetypal contents that can respond to the profiles of erotic leader (who “brings out and reflects back the healthy self-love and self-admiration that exists in everyone”), trickster-as-leader (producing attraction toward his/her visionary ideas), and sibling leadership (establishing alliances through decentralization and shared work in networks).

The training of future leaders based on the psychological types employed must also take gender differences into account; thus, equality between men and women – in addition to responding to quotas in forming teams – would be complemented by the diversity stemming from incorporating other psychological types to develop teamwork. In a more general sense, the data obtained corroborate the usefulness of the MBTI in the study of leaders (Brown and Reilly, 2009; Mattare, 2015) or students similar to those in our sample (Ribeiro Filho et al., 2010; Bak, 2012; Mohammadi et al., 2018).

When analyzing the stories in which the participants were the protagonists, the use of the contents established by Campbell (1949/2004) revealed a basic structure of the heroic when carrying out this active imagination activity. Specifically, the majority of the sample offered stories in which the hero / heroin was confronted with a mystery to solve (or mission to fulfill), faced difficulties, and, finally, achieved harmony between the personal and the collective. The stronger presence of these basic contents encourages reflection about the need to make other contents visible that can be present in one’s image of heroism; for example, by being aware of the transformations that can take place or the distractors that can appear while carrying out the mission itself. Additionally, another element that can help to make our inner hero / heroine visible is the incorporation of the archetypal images as energizers of our psyche (as in studies like those by Sanders and van Krieken, 2018), as well as the epistemic and the energizing functions that the hero stories fulfill (Allison and Goethals, 2014).

Considering values as an interstitial concept between the collective and the individual has revealed the importance, in the group studied, of both individualist (Self-direction) and collectivist (Benevolence) values – with women significantly more oriented toward two of the collectivist values (Conformity, Tradition) and one of the mixed-type values (Universalism) (Schwartz et al., 2001; Abella García et al., 2017). These gender differences also suggest incorporating personal values as another element to take into account when analyzing the profiles of leaders, especially because differences were observed based on the main characteristics of the psychological types and the content of the story about their own hero / heroine (particularly those who wrote a Return in which they reconciled the individual and the universal). In sum, the set of instruments used in this study appears to be useful for future studies, given the information they provide about the profile of leaders, their imaginary hero, and the values that orient them.

## Limitations and Future Agenda

The findings of this study must be considered in light of its limitations. The size of the sample studied and the procedure through which the participants joined the study partly restrict the explanatory power of the results, pointing to the need for statistical controls – in addition to the employees – in the research process. This will positively affect the comparison of our data with those from broader studies at a more collective level. Regarding the individual level, future studies will have to include the process of raising consciousness about one’s own heroism in leadership – and how heroism is integrated into people’s daily lives.

This awareness is susceptible to being investigated through a longitudinal study, taking into account that the entire process of self-awareness has fluctuations that can influence the stability of the measures. In this regard, the instruments used must respond to both an adequate statistical functioning and ontological coherence in its use, that is, making the measures serve the underlying theory of the study – rather than the contrary. As an example of this reflection, future applications of the MBTI in longitudinal studies must take into account the individuation process of those who respond– in the direction of what was described by Myers (2016) – and the need for greater dialog between this instrument and others, such as the FFM – as mentioned in the studies by Lloyd (2015) or Cooper et al. (2017).

In a more general sense, this exploration of the heroic element of leadership is consistent with contributions linked to the so-called *moral modeling* or *moral career*, that is, by offering guidelines that influence the way one might lead his/her own life (Allison and Goethals, 2015; Kinsella et al., 2017; Nakamura and Graham, 2017; Walker, 2017). This task itself – which is heroic – will take shape, represented by establishing the bases of heroic wellbeing (Efthimiou et al., 2018; Williams, 2018), and reminding us that every life transition has the potential to be transcendent.

## Ethics Statement

All subjects gave written informed consent prior to the collection of the research data. The ethical requirements of the Ethics Committee of the University of Barcelona were applied to the current study, which meant that additional approval for the research was not required because the data obtained did not involve animal or clinical experimentation. Additionally, this study complies with the recommendations of the General Council of Spanish Psychological Associations (Consejo General de Colegios de Psicólogos) and the Spanish Organic Law on Data Protection (15/1999: Jefatura del Estado, 1999).

## Author Contributions

JP conceived and designed the research, drafting the work, and revising it critically for important intellectual content. NC was responsible for the analysis and interpretation of data gathered during the research, revising it critically for important intellectual content.

## Conflict of Interest Statement

The authors declare that the research was conducted in the absence of any commercial or financial relationships that could be construed as a potential conflict of interest.
